# Mycophagous beetle females do not behave competitively during intrasexual interactions in presence of a fungal resource

**DOI:** 10.1002/ece3.8977

**Published:** 2022-06-02

**Authors:** Lisa D. Mitchem, Vincent A. Formica, Reena Debray, Dana E. Homer, Edmund D. Brodie

**Affiliations:** ^1^ 2358 Mountain Lake Biological Station and Department of Biology University of Virginia Charlottesville Virginia USA; ^2^ Department of Biology Swarthmore College Swarthmore Pennsylvania USA; ^3^ Department of Integrative Biology University of California Berkeley California USA

**Keywords:** *Bolitotherus cornutus*, coleoptera, competition, exclusion, female contest, female–female interactions

## Abstract

Intrasexual interactions can determine which individuals within a population have access to limited resources. Despite their potential importance on fitness generally and mating success especially, female–female interactions are not often measured in the same species where male–male interactions are well‐defined. In this study, we characterized female–female interactions in *Bolitotherus cornutus*, a mycophagous beetle species native to Northeastern North America. We used dyadic, behavioral assays to determine whether females perform directly aggressive or indirectly exclusionary competitive behaviors. Polypore shelf fungus, an important food and egg‐laying resource for *B*. *cornutus* females, is patchily distributed and of variable quality, so we tested for competition over fungus as a resource. Behavior of females was assessed in three sets of dyadic trials with randomly paired female partners. Overall, females did not behave aggressively toward their female partner or perform exclusionary behaviors over the fungal resource. None of the behaviors performed by females were individually repeatable. Two scenarios may explain our lack of observed competition: our trial context may not induce competition, or female *B*. *cornutus* simply may not behave competitively in the wild. We compare our results to a similar study on male–male interactions in the same species and propose future studies on female–female interactions under different competitive contexts to expand the understanding of female competition.

## Graphical abstract

Male competition is described in many species while the intricacies of female–female interactions are less known, despite the fact that both processes are important functions of sexual selection. In this study, we used dyadic, behavioral assays to determine whether female *Bolitotherus cornutus* perform directly aggressive or indirectly exclusionary competitive behaviors. Overall, females did not behave aggressively toward their female partner or perform exclusionary behaviors over the fungal resource.

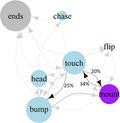

## 1 INTRODUCTION

Individuals, male or female, should engage in intrasexual competition when resources are limited or variable (Arnocky et al., 2014; Baniel et al., 2018; Knell, 2009; Rosvall, 2011), but research on intrasexual behaviors is biased toward studies on males. Male competition is described in many species while the intricacies of female–female interactions are less known, despite the fact that both processes are important functions of sexual selection (Candolin, 1999; Hunt et al., 2009; Moore & Moore, 1999; Wong & Candolin, 2005; Zhu et al., 2016). There is no reason to expect females to also compete over resources because interactions among females can affect which individuals have access to resources and ultimately lead to selection on behaviors exhibited in agonistic and competitive interactions (Cain & Langmore, 2016; Clutton‐Brock, 2009; Goubault et al., 2007; Hare & Simmons, 2019; Rosvall, 2011). Characterizing and defining female–female interactions is critical for ultimately determining how individuals distribute themselves within a specific habitat and how specific behavioral phenotypes affect resource allocation (Stockley & Campbell, 2013).

Female competition typically has not been characterized in species in which male competition is well‐defined (Hunt et al., 2009; Stockley & Bro‐Jørgensen, 2011). In insects, female competition is largely unexplored, despite considerable work on male competition (Dunn et al., 2015; Giron et al., 2004; Goubault et al., 2007; Kaiser et al., 2019; Kemp & Wiklund, 2001). In fact, research on female competition comes largely from mammals (Arnocky et al., 2014; Baniel et al., 2018; Haunhorst et al., 2020; Stockley & Bro‐Jørgensen, 2011) and birds (Cain & Ketterson, 2013; Cain & Langmore, 2016; Thys et al., 2017). The bias toward characterizing male competition is largely due to a subset of species in which males display overtly aggressive interactions because overt physical competition is easily observed and therefore easily characterized (Berglund et al., 1996; Holekamp & Strauss, 2016; Kemp & Wiklund, 2001; Tinghitella et al., 2018). Conversely, female competition is often described as more lengthy or discrete (Clutton‐Brock, 2007, 2009; Čokl et al., 2020; Stockley & Campbell, 2013), though this classification may reflect human biases and not direct quantification of female aggressiveness (Kamath & Wesner, 2020; Rubenstein, 2012).

Objectivity is essential for initial studies of behaviors in a species where previous research cannot provide insight on potential consequence of interactions. Competition behaviors, in particular, can be difficult to compare between sexes because they are categorized based on observational studies and the observer risks imparting their own judgment on the intention of those behaviors (Burghardt et al., 2012; Tuyttens et al., 2014). Objectivity can be achieved using a strict but inclusive definition of aggressiveness for both sexes. Aggression is broadly defined as any behavior that intimidates or harms a social partner to an extent that causes them to flee the immediate area (Holekamp & Strauss, 2016). The base observation that one individual leaves the area can therefore be used as a means of categorizing behaviors, where any behavior that leads to an individual immediately fleeing is determined to be aggressive in nature (Mitchem et al., 2019). Mitchem et al. (2019) constructed an ethogram of male–male interactions using contingency analysis where only the significant transitions from one behavior to the ending of an interaction were considered aggressive. Using this definition, all behaviors utilized during same‐sex interactions can be observed and characterized without unintended human bias. Moreover, measuring same‐sex interactions using the same behavioral paradigm for both males and females is critical for determining the meaning of those behaviors.

Understanding the proportion of behavior that is attributed to differences among individuals is essential for ultimately determining the consequences of behavioral interactions. Despite high plasticity, behaviors often have a component of variance that remains repeatable from one context to the next (Kralj‐Fišer & Schuett, 2014; Sih et al., 2004), and it is this repeatable proportion of a behavior that is attributable to intrinsic differences among individuals providing a fixed phenotype that may be subject to sexual selection (Réale et al., 2007; Schuett et al., 2010; Wolf & Weissing, 2012). Behaviors that are not repeatable may show no response to selection because their plasticity across contexts equalizes fitness outcomes for all individuals expressing that behavior (Boake, 1989; Schuett et al., 2010). Therefore, estimating repeatability is an important first step to understanding evolvability of behavioral traits.

In this study, we tested if female forked fungus beetles (*Bolitotherus cornutus*) compete aggressively over a food and egg‐laying resource. We characterized and quantified their physical interactions and then tested for exclusionary competition by quantifying the proportion of time females monopolized the provided resource over their same‐sex partner. We also tested if female social interactions were repeatable. We use the same behavioral trial paradigm as a similar study on male–male competitive behaviors in this species (Mitchem et al., 2019) and argue for the importance of measuring male‐male and female–female behaviors equally.

*Bolitotherus cornutus* is a sexually dimorphic, mycophagous beetle species native to Northeastern North America (Liles, 1956). *B*. *cornutus* are found feeding and interacting on three species of polypore shelf fungus—*Ganoderma tsugae*, *Ganoderma applanatum*, and *Fomes fomentarius*—that grow on dead logs in forested habitats: (Liles, 1956). Fungus quantity and quality is variable both within and across populations (L. Fornof, E.D. III Brodie, V.A. Formica, unpublished data). Documented social interactions including male–male competition, mating, and egg laying occur on these fungus shelves (Conner, 1988; Liles, 1956; Pace, 1967). While territoriality is not documented in *B*. *cornutus*, males often engage in combat while on the fungal shelves even when no females are present (Mitchem et al., 2019), and have been observed walking along the periphery of the shelves in a potentially patrolling‐like manner (pers obs.). Beetles mate and females lay eggs continuously throughout their active season (mid‐May to mid‐October) (Pace, 1967). Once females lay eggs on the fungus, larvae hatch inside the fruiting body and consume it until they eventually emerge as adults (Liles, 1956; Pace, 1967). Larval fitness differs depending on the fungus species they develop in, but females may be limited in their movement among populations as they are more likely to lay eggs on lower quality fungus than migrate (Wood et al., 2014). Larval competition is common within fungal brackets, as larvae often cannibalize each other as they develop within their fungus (Liles, 1956; Wood et al., 2014), so females may compete over fungal resources to maximize offspring survival.

Male competitive behaviors are well characterized in *B*. *cornutus* (Mitchem et al., 2019), but less is known about how females interact and whether they compete over resources. Males are distinguished by their two sets of horns, clypeal and thoracic, whereas females are hornless but have two small tubercles on the top of their pronotum where horns would be. Males use both sets of horns in competitive interactions to gain access to mates by prying courting or mating males off the backs of females (Brown et al., 1985). Males also engage in combat over access to fruiting bodies with no females present, and both aggressive and nonaggressive behaviors in this context are highly repeatable (Mitchem et al., 2019). Males who win competitive interactions gain more access to females who allow males to passively court but can block attempted copulation by closing their anal sternite (Brown et al., 1985; Conner, 1988). While past field observational studies did not detect the presence of competitive behaviors in females (Conner, 1988; Formica et al., 2012; Liles, 1956), variable mate quality (Conner, 1988, 1995), patchy resource quality within populations (Fornof et al., in prep), and larval cannibalism (Liles, 1956; Wood et al., 2014) all provide motive for female–female competition.

## 2 METHODS

### 2.1 Beetle collection and morphological measurements

We collected beetles from a large metapopulation near Mountain Lake Biological Station in Pembroke, Virginia (May 2016). We housed 47 female beetles in natural light conditions with temperature held constant at 20 ± 1.5°C. Beetles were isolated in 5 × 2.5 × 5 cm, plastic containers for one month before we conducted trials. Beetle containers consisted of plaster as a substrate to retain moisture, mulch, and a piece of *Ganoderma tsugae* fungus as food. We provided water to beetles as needed.

Following collection, we imaged beetles using a flatbed Scanner (Epson Perfection V600 Photo) and used those images to measure beetle elytra length to the nearest 0.01 mm in ImageJ (Abramoff et al., 2004). We then assigned each beetle a unique ID and painted a white or black stripe along the sides of both elytra using nontoxic Testors^®^ Enamel paint so we could differentiate between individuals in trials.

### 2.2 Female–female interaction trials

We performed dyadic, female–female interaction trials in July 2016 following methods from Mitchem et al. (2019). Each trial consisted of two beetles interacting freely in a small plastic container (10 × 10 cm) filled approximately 2 cm deep with plaster for four hours. We provided an embedded, 5 × 5 cm square of *G*. *tsugae* in the trial containers (hereafter referred to as arenas) as a resource for females to fight over. Because *B*. *cornutus* are most active at night, we conducted trails in a dark, temperature‐controlled room held at 19 ± 2℃. A Canon PowerShot G1 X digital camera on infrared setting placed 1 m above the arenas recorded female–female interactions trials by taking snapshot images every 5 s for 4 h. We controlled the camera's shutter speed using Neewer© LCD digital shutter release remote control.

A total of 47 females were paired in three different combinations of female–female interaction trials (71 trials total). We paired each female randomly with respect to body size but always paired females painted white with females painted black to differentiate between individuals in each trial. We returned females to their isolated housing containers after each trial and waited two days before any female was re‐paired in a new trial.

To conduct behavioral observation of female–female interactions, we first stitched still images from each trial into time‐lapse videos using FFmpeg software (version be1d324). Behavioral observations were completed by DEH and LDM, who scored the initiation and duration of the following behaviors (described in Mitchem et al., 2019): touch, bump, head, mount, grapple, chase, flip, and end. LDM and DEH trained together to ensure interobserver consistency in scoring of specific behaviors. We scored whenever a female came within proximity of her partner, which was scored as approaching to at least one body length of their partner without physically touching. Fungus patrolling, or the duration of time each beetle spent alone on the fungal resource, was quantified to determine if females perform any resource guarding or exclusionary competition behaviors. We remained objective about which behaviors were considered aggressive and nonaggressive and later used ethogram analysis to determine specific classifications for each behavior (see Section 2.3).

### 2.3 Ethogram construction

Following Mitchem et al. (2019), we constructed an ethogram containing statistically significant transitions among behaviors to describe the most probable sequences of interactions in female–female trials. To create the female–female ethogram, we first constructed a matrix of transitions among all behaviors in the first set of trials (*N* = 23 interaction trials). We then combined all trial matrices into one matrix representing the total number of transitions among behaviors for every female's first trial. The final matrix was tested using a contingency analysis to determine which transitions occurred at a greater likelihood than expected by random chance. We constructed the ethogram of probabilities of transitions among behaviors using Markov chain analysis on the final matrix (R package: markovchain (Spedicato, 2017)). We used only the first set of trials to avoid pseudoreplication that would have been caused by using data from each female three times. Results did not differ based on which trial set we used, and using the first allowed us to directly compare the female–female ethogram to the related male–male study, Mitchem et al. (2019), that also used the first set of trials.

Once ethograms were constructed, we characterized behaviors into categories based on their transitions with other behaviors. We followed the rationale used by Mitchem et al. (2019) to define male–male competitive behaviors as either nonaggressive, aggressive, or mounting. Nonaggressive behaviors were more likely to lead to another behavior, aggressive behaviors most frequently resulted in the ending of an interaction, and mounting behaviors occur when one individual climbs on top of their partner's back (Holekamp & Strauss, 2016; Mitchem et al., 2019). Mounting behaviors are given their own specific designation because they were previously assumed to only occur in male–female courtship contexts, but were frequently observed in male–male competition (Mitchem et al., 2019) and our female–female competition trials (see Section 3).

### 2.4 Statistical analysis

We calculated intraclass correlations (ICCs) to quantify within‐individual repeatability of each observed behavior. ICCs require measurements of within‐individual variance and between‐individual covariance. We obtained these measurements of variance and covariance for each behavior from univariate linear mixed models implemented in a Bayesian framework. Using the “MCMCglmm” package in R (Hadfield, 2010), our models included individual behaviors (touch, bump, head, etc.) as the dependent variables and female ID and trial ID as random effects. Our MCMC analysis included 500,000 iterations, thinning intervals of 100, and a burn‐in rate of 5000. We used noninformative priors with an assumed Poisson error model. MCMCglmm outputs variance components for fixed effects, random effects, and residuals. We used the variance outputs of our random effects, female ID, and residual variance to calculate ICCs. We created six total models—one model for each behavior observed in our female–female interaction trials.

Next, we assessed the effects of body size and interactive behaviors on access to the provided fungal resource. Only 23 of our females performed any fungus patrolling behavior, so we scored fungus patrolled as a binomial factor where females were categorized as either performing any fungus patrolling behavior (*n* = 23), or performing no fungus patrolling behavior (*n* = 24). We used a *T*‐test to assess differences in body size and Mann–Whitney *U* test to assess differences in interactive behavior (# of initiated behaviors by that female in a trial) between females who did and did not perform fungus patrolling behaviors. To avoid pseudoreplication, we selected the first trial for each female to be represented as their score for interactive behaviors in our final analysis. We used a Mann–Whitney *U* test for our second analysis because our behaviors did not fit a normal distribution. We performed our *T*‐test and Mann–Whitney *U* test in R v.3.6.0.

## 3 RESULTS

**FIGURE 1:**
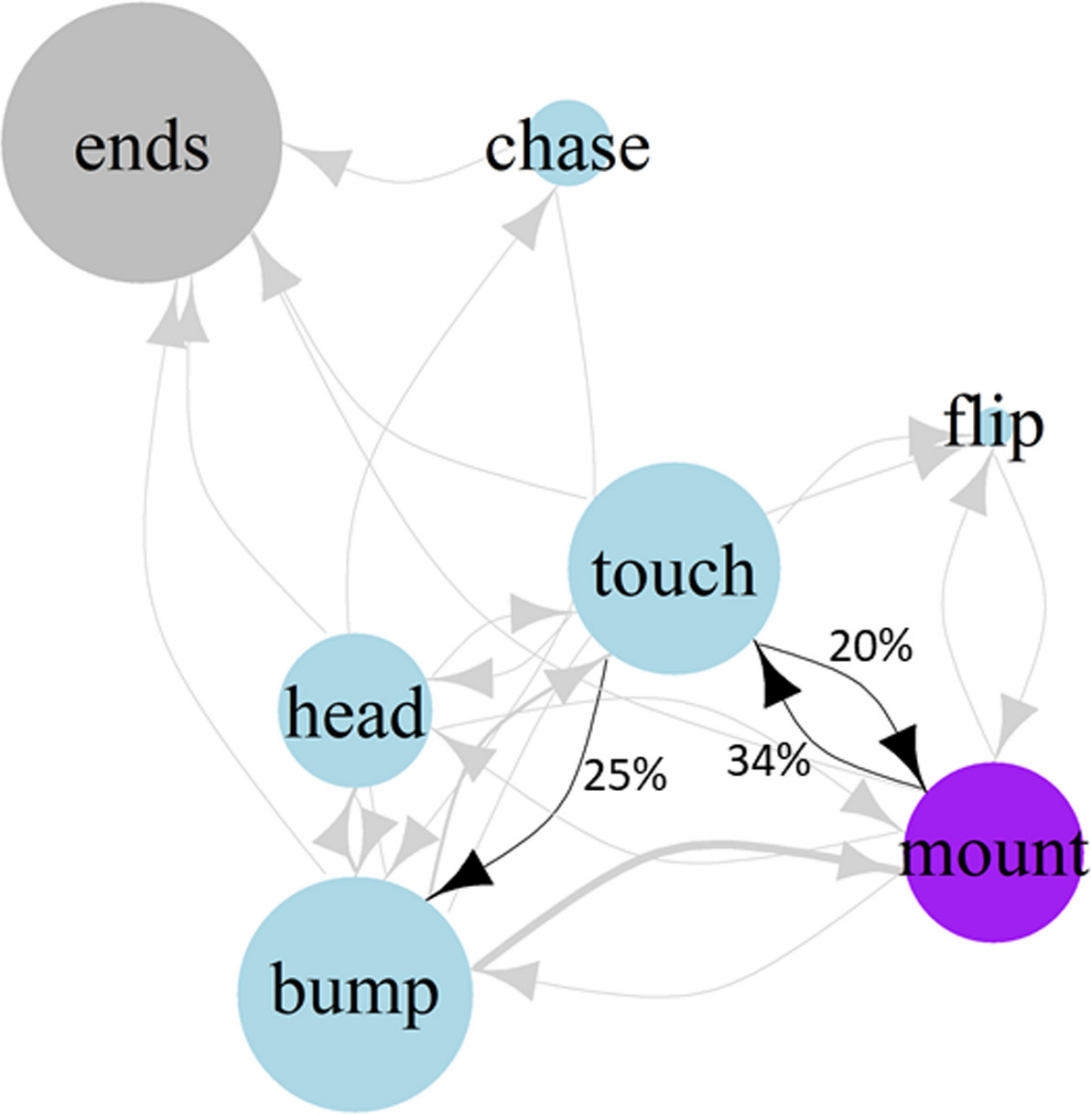
Ethogram for female–female interactions. Size of the circle indicates the relative number of times a behavior occurred across all trials. Colors signify the type of behavior where blue is nonaggressive and purple is mounting. Arrow width indicates the probability a behavior transitioned to the next behavior where significant transitions are labeled black and nonsignificant transitions are labeled gray. Specific probabilities are noted on the arrow line of significant transitions

Females spent an average of 3.1% (range: 0–47.8%) of their 4‐hour trial period interacting with their female partners. No behaviors in female–female interaction trials were repeatable across the three trials (Table 1). Almost half of all initiated interactions (48%) consisted of females coming within proximity of their partner, and then ending the interaction without engaging in any physical contact. The most common physical interactions included touching, bumping, mounting, and head‐to‐head. Females frequently engaged in the bumping behavior (32.6% of initiated physical behaviors) and often cycled through bouts of touching and mounting (Figure 1). Females performed a small frequency of head‐to‐head (9.7%), chases (3.5%), and flips (1.1%), though these behaviors did not have any statistically supported transitions to other behaviors (Figure 1). Based on our definition of aggressiveness, we could not characterize any females’ behaviors as either aggressive or nonaggressive. No behaviors transitioned to ending the interaction more frequently than to other behaviors.

**TABLE 1:** Repeatability (intraclass correlation coefficient) measurements for behaviors measured in female–female interaction trials. Bracketed values represent 95% lower and upper HPD intervals

Behavior	Description (Mitchem et al., 2019)	Female ICC
Touch	Any physical contact that is not characterized by another behavior	0.001 [0.00, 0.22]
Mount	One beetle crawls onto the back of the second beetle	0.001 [0.00, 0.17]
Bump	Head of one beetle comes into contact with any part of the body of the second beetle	0.003 [0.00, 0.43]
Head	Both beetles touch head to head	0.001 [0.00, 0.39]
Chase	One beetle rapidly follows the second beetle	0.002 [0.00, 0.68]
Flip	One beetle flips the second beetle onto its back	0.003 [0.00, 0.90]

Fungus patrolling duration averaged 8.91 min [range: 0.00–218.95 min] among all female–female interaction trials. Females performed fungus patrolling behaviors in 17 of our 71 trials. A total of 23 females performed fungus patrolling behaviors in any of their three female–female interaction trials while 24 females did not patrol the fungus squares. Females who performed fungus patrolling behaviors did not differ in body size (*t* = 1.00, *df* = 45.11, *p* = .32) or total interactive behaviors (*w* = 275, *p* = .99) from females who performed no fungus patrolling behaviors.

## 4 DISCUSSION

Female–female interactions in *B*. *cornutus* were neither overtly aggressive nor exclusionary in our study. Instead, females only interacted with their female partner 3% of the time and spent a majority of their interactive time either in close proximity of their female partner or cycling through bouts of bumping, touching, and mounting. No female behaviors were individually repeatable, so behaviors in our trials may be driven more by extrinsic variation in social and abiotic environments, including characteristics of social partners or environmental differences among trials. Behaviors that were categorized as aggressive in male–male interaction trials—grapple, chase, and flip (Mitchem et al., 2019)—were not categorized as aggressive in our female–female interaction trials.

When studying female–female interactions, it is important to remain objective in the classification of behaviors. Both sexes should be tested for the presence of competition using the same guidelines to limit unintended observer bias. Classification of female competition as exclusionary or absent may be due to our own human bias in what we expect from behavior in different sexes (Kamath & Wesner, 2020; Rubenstein, 2012). Our lab assays allowed us to parse apart the individual behaviors performed by females and analytically determine if any of those behaviors were aggressive. Using our method, we were able to objectively test females in the same way as males but categorize behaviors differently by observing the interactions immediately following each behavior. We determined that some behaviors have different functions when performed by males versus females when using the same criteria to evaluate them. Both males and females perform chasing and flipping behaviors, but these behaviors appear to be aggressive in males (Mitchem et al., 2019) and nonaggressive in females. Though only accounting for 16% of initiated behaviors, male aggression under the same context as our female trails was highly repeatable (ICC of 0.8, Mitchem et al., 2019). Nonaggressive behaviors in males were also highly repeatable (ICC of 0.4, Mitchem et al., 2019), whereas no female behaviors were repeatable. Males also performed more interactions with their partners for a longer amount of time compared to females (males: 10.3% of trial time, females: 3.1% of trial time) (Mitchem et al., 2019).

Two possible scenarios may explain the lack of competition between females in our trials. First, competition is context specific (Clutton‐Brock, 2009; Giron et al., 2004; King, 1989), and our trial context may not induce competition in female *B*. *cornutus*. Competition occurs when valuable resources at that given moment are limited or vary in quality within an environment (Clutton‐Brock, 2009; Rosvall, 2011). If females in our trials were not physiologically ready for oviposition, then our provided egg‐laying resource would be superfluous. Females in our experiment were isolated for a short period of time prior to behavioral trials and had little time to assess the trial environment, which could affect motivation for competition in our trials. The specific context is also likely important for female–female competition. Females may be more likely to engage in aggressive behaviors when mates are the limiting resource (reviewed in: Rosvall, 2011), or resources are highly variable in quality (Cain & Ketterson, 2013; Elias et al., 2010; Stockley & Campbell, 2013). We also found no evidence for indirect or exclusionary competition, which is more often observed when food resources are scarce (Rosvall, 2011). The quantity of fungus provided in our study may not be resource limiting for females.

A second explanation for our lack of observed competition may be that female *B*. *cornutus* simply may not behave competitively toward one another in the wild. Based on analysis of *B*. *cornutus* social networks, female–female interactions do not correlate with fitness effects even when resource distribution was manipulated (R. A. Costello, P. A. Cook, V. A. Formica, E. D. III Brodie, unpublished data). Our results also support previous field studies that found, while male aggression is often observed in the wild, *B*. *cornutus* females do not engage in similar aggressive behaviors (Conner, 1989; Formica et al., 2012; Pace, 1967). If food, mates, and egg‐laying resources are not limited, then we would expect competition and aggression to negatively affect fitness in females (Cain & Ketterson, 2013; Stockley & Campbell, 2013). Although fungal resources vary among wild *B*. *cornutus* populations (Fornof et al., in prep), that variation may not reflect meaningful differences in quality and abundance for females.

One of the more surprising results in our study was the lack of repeatability in any specific behavior of females. Both aggressive and nonaggressive behaviors in male–male forked fungus beetle interactions are highly repeatable (Mitchem et al., 2019), which led us to predict similar levels of repeatability for those behaviors in females. The lack of repeatability observed in females, however, aligns with what is already known about female–female interactions in other species. Females may be more plastic in their response to intrasexual stimuli compared to males (Stockley & Campbell, 2013). Behavioral plasticity in response to potential competition is a more efficient strategy for females who require a greater energetic cost of gamete production (Clutton‐Brock, 2007; LeBas, 2006; Stockley et al., 2013). Regulating costly aggressive behaviors depending on the context allows females to invest more in egg quality and/or quantity (Stockley et al., 2013). Alternatively, partner behaviors may have been too variable and therefore unable to elicit the same response from trial to trial (Dingemanse & Dochtermann, 2013; Dingemanse et al., 2010). High variance in social partner behavior followed by variable focal female response would result in higher within‐individual variance and overall lower repeatability (Nakagawa & Schielzeth, 2010; Wolak et al., 2012).

## 5 CONCLUSION

Future studies should measure female–female interactions in different contexts including presence/absence of a male cue, and variation in fungal resource quality or quantity to further elucidate potential competitive behaviors. Measuring females under multiple contexts will aid in determining the level of plasticity for these female–female interactive behaviors both within a specific context, as demonstrated in our present study, and across contexts. Overall, we show that female and male intrasexual interactions have similar social behaviors, but these behaviors differ in their elicited responses. Using the same behavioral paradigm for both sexes allowed for direct comparison of same‐sex interactions and objective, unbiased quantification of behaviors.

## AUTHOR CONTRIBUTIONS

**Lisa D. Mitchem:** Data curation (equal); Formal analysis (lead); Investigation (equal); Methodology (equal); Project administration (equal); Validation (equal); Visualization (lead); Writing – original draft (lead); Writing – review & editing (equal). **Vincent A. Formica:** Conceptualization (equal); Data curation (equal); Formal analysis (supporting); Funding acquisition (equal); Investigation (equal); Methodology (equal); Resources (equal); Supervision (equal); Writing – review & editing (equal). **Reena Debray:** Conceptualization (equal); Data curation (lead); Investigation (equal); Methodology (lead); Project administration (equal); Writing – review & editing (supporting). **Dana E. Homer:** Data curation (equal); Methodology (supporting); Writing – review & editing (supporting). **Edmund D. Brodie III:** Conceptualization (supporting); Funding acquisition (lead); Investigation (supporting); Project administration (supporting); Resources (equal); Supervision (equal); Validation (equal); Writing – review & editing (equal).

## CONFLICT OF INTEREST

The authors declare no competing interests.

## Data Availability

All data and relevant R code used are archived in Dryad Repository at https://datadryad.org/stash/share/5lH33LpOGa‐4vxFphbs4OoEhtn8L6b_PC‐JOwz_3NDQ.
